# Progressive effect of beta amyloid peptides accumulation on CA1 pyramidal neurons: a model study suggesting possible treatments

**DOI:** 10.3389/fncom.2012.00052

**Published:** 2012-07-23

**Authors:** Viviana Culmone, Michele Migliore

**Affiliations:** ^1^Institute of Biophysics, National Research CouncilPalermo, Italy; ^2^Department of Mathematics and Informatics, University of PalermoPalermo, Italy

**Keywords:** Aβ-peptide, hippocampal neuron, realistic model, ion channels modulation

## Abstract

Several independent studies show that accumulation of β-amyloid (Aβ) peptides, one of the characteristic hallmark of Alzheimer's Disease (AD), can affect normal neuronal activity in different ways. However, in spite of intense experimental work to explain the possible underlying mechanisms of action, a comprehensive and congruent understanding is still lacking. Part of the problem might be the opposite ways in which Aβ have been experimentally found to affect the normal activity of a neuron; for example, making a neuron more excitable (by reducing the *A*- or *DR*-type *K*^+^ currents) or less excitable (by reducing synaptic transmission and *Na*^+^ current). The overall picture is therefore confusing, since the interplay of many mechanisms makes it difficult to link individual experimental findings with the more general problem of understanding the progression of the disease. This is an important issue, especially for the development of new drugs trying to ameliorate the effects of the disease. We addressed these paradoxes through computational models. We first modeled the different stages of AD by progressively modifying the intrinsic membrane and synaptic properties of a realistic model neuron, while accounting for multiple and different experimental findings and by evaluating the contribution of each mechanism to the overall modulation of the cell's excitability. We then tested a number of manipulations of channel and synaptic activation properties that could compensate for the effects of Aβ. The model predicts possible therapeutic treatments in terms of pharmacological manipulations of channels' kinetic and activation properties. The results also suggest how and which mechanisms can be targeted by a drug to restore the original firing conditions.

## Introduction

Alzheimer's Disease (AD) is one of the most common neurodegenerative disorders associated with memory deficits and cognitive decline leading to elderly dementia (Selkoe, 1999; Brookmeyer et al., 2007). It is well known that it mainly involves structural alterations in the CA1 region of hippocampus (Goedert, 1987; Hasselmo, 1997; Schenk et al., 1999) and it is characterized by neuronal loss, intracellular neurofibrillary tangles (NFT), and extracellular neuritic plaques (Nagy et al., 1995), whose major constituent are Aβ peptides (Iwatsubo et al., 1994), secreted after cleavage of a single transmembrane precursor, termed amyloid precursor protein (APP). Despite persistent research efforts to understand the molecular events leading to AD, the causes of the disease are not understood. Several aspects have been implicated in AD pathogenesis. Mechanisms such as synaptic loss (Hamos et al., 1989; Terry et al., 1991; Terry, 2000; Knobloch and Mansuy, 2008), mitochondria dysfunction (Moreira et al., 2001; Manczak et al., 2006; Eckert et al., 2008; Reddy and Beal, 2008), network instability (Palop and Mucke, 2010), disregulation in calcium homeostasis (Mattson and Chan, 2001), alterations of some ionic channels, astrocytic response to plaque deposition (Kuchibhotla et al., 2009), amyloid angiopathy (Rensink et al., 2003), and alteration in the function of N-methyl-D-aspartic acid (NMDA) receptors (Snyder et al., 2005; Shankar et al., 2007; Sato et al., 2008; Texidó et al., 2011) have all been reported to be affected by Aβ deposition on neuronal membrane. However, it is very difficult to understand the roles, interactions, and contribution of these disparate mechanisms to the overall picture of the disease. A partial source of confusion could be that experimental studies are usually carried out to isolate the effects of a single mechanism, and/or on transgenic mouse models with unknown compensatory effects. This may lead to a set of results that could be conflicting (Good et al., 1996; Plant et al., 2006; Rui et al., 2006; Kerrigan et al., 2008; Kloskowska et al., 2008) or difficult to interpret.

In the present study, we were interested in investigating how modifications of the synaptic and membrane properties caused by Aβ accumulation can affect the main firing properties of a neuron. To this purpose, we used a realistic computational model of a hippocampal CA1 pyramidal neuron including the electrophysiological effects of Aβ on neuronal membrane, modeled according to different independent experimental studies. With this model, we were able to make experimentally testable predictions on the contribution of each mechanism to the overall modulation of the cell's excitability, and point out possible targets for therapeutic treatments that could be used to restore the original firing conditions.

## Materials and methods

All simulations were implemented using v7.2 of the NEURON simulation environment (Hines and Carnevale, 1997). Most simulations were carried out on a parallel computer system using up to 200 processors (IBM Linux cluster, CINECA, and Bologna). The model and simulation files specifically used for this work are available for public download under the ModelDB section of the Senselab database (http://senselab.med.yale.edu).

In all simulations we used the cell 5038804, a hippocampal CA1 pyramidal neuron originally downloaded from the public archive www.neuromorpho.org, composed by 173 membrane sections modeled with 559 segments. The set of passive properties, voltage-dependent ionic channels, and their kinetics and distribution were identical to those in (Migliore et al., 2008, ModelDB a. n.87535). In this model, already validated against a number of different experimental findings on electrophysiological and synaptic integration properties of CA1 neurons (e.g., Migliore, 2003; Gasparini et al., 2004), sodium (*I*_*Na*_) and delayed rectifier potassium (*K*_*DR*_) conductances were uniformly distributed throughout the dendrites, whereas *K*_*A*_ and *I*_*h*_ conductances linearly increased with distance from soma.

**Figure 1:**
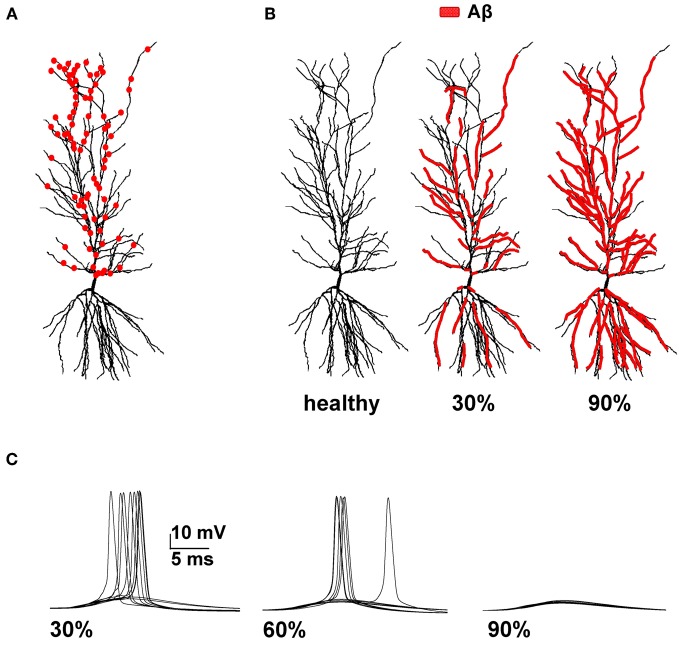
**Model setup. (A)** Schematic representation of the hippocampal CA1 pyramidal neuron used in all simulations (cell 5038804 in the neurophorpho.org public archive). Red circles represent a typical distribution of the 100 excitatory synapses, randomly redistributed and activated during each simulation, used to calculate the average spike probability; **(B)** Aβ-peptides progressive accumulation was modeled by modifying the intrinsic active and synaptic properties of increasing membrane area; the panels represent the control conditions *(left, healthy)* and typical cases where 30% (*middle*) or 90% (*right*) of membrane was affected by Aβ. **(C)** Typical somatic membrane potential at three different stages of disease (30–60–90% of impaired membrane area) during activations of synaptic inputs (1.20 nS). Traces show the results for 10 different simulations using a random redistribution of synaptic locations, affected dendrites and synaptic activation times. Note the lower number of spikes (i.e., lower spike probability) as the membrane area affected by Aβ increases.

To model synaptic activation, two sets of 50 AMPA excitatory synapses (modeled as a double exponential conductance change with 0.5 and 3 ms for rise and decay time, respectively) were randomly distributed in the distal (>300 μm from soma) and in the proximal apical region (Figure 1A). We used the same peak synaptic conductance for all proximal synapses, testing several different values in the range 0.9–2 nS. To take into account experimental findings, distal synapses were three times weaker than proximal ones (Megìas et al., 2001). Their activation time was drawn from a Gaussian distribution with a standard deviation of 5 ms (corresponding to the positive sweep of a γ-cycle), and the distal set of synapses were activated with a 5 ms delay with respect to the proximal ones [to take into account the physiological delay between the Perforant Path and Schaeffer Collaterals pathways, see Migliore (2003)]. Average control conditions for the spike probability of the neuron were calculated from 100 simulations, randomly redistributing synaptic locations and activation times. NMDA and inhibitory synapses were not included in the model. For NMDA synapses, their properties and kinetics suggest that they would not change the qualitative results obtained for the relatively simple stimulation protocol used in our simulations, i.e., single synaptic activations. For inhibitory synapses, there is not enough information on the effects of Aβ on the interneurons responsible for feedforward inhibition to implement a reasonable model. A more detailed analysis of their actual role under our stimulation conditions was thus considered outside the scope of this paper.

To model Alzheimer effects, randomly chosen sections of membrane composing a given portion of the total somato-dendritic membrane area were selected, as schematically represented in Figure 1B. Their properties were then modified according to experimental findings: in those compartments, *K*_*A*_ current peak conductance was reduced by 60% (Good et al., 1996), *K*_*DR*_ by 40% (Good et al., 1996), and *Na* by 50% (Kim et al., 2007), whereas the peak synaptic conductance of any input targeting an affected compartment was decreased by 50% (Kamenetz et al., 2003; Parameshwaran et al., 2007; Wei et al., 2010; Perez-Cruz et al., 2011). At each stage of the disease (0–100% of affected membrane), the spike probability was calculated from a set of 100 simulations, randomly redistributing the affected compartments, synaptic distribution, and activation times.

It may be questioned that we did not implement the effects of Aβ on calcium channels. The reason for this choice was that experimental findings in this case are somewhat contradictory and unclear. As observed by Rui et al. (2006) most of the confusion could be due to the fact that different kinds of Aβ fragments (e.g., Aβ_25−35_, Aβ_1−40_, or Aβ_1−42_) could affect in different ways the various cellular mechanisms involved in calcium regulation (different types of Ca^2+^ channels or pumps). For example, they observed that Aβ_1−42_ inhibits Ca^2+^ oscillations. However, Kloskowska et al. (2008) showed, in hippocampal neurons from an AD transgenic rat, that high levels of Aβ can instead increase their frequency with respect wild-type neurons. Nevertheless, we carried out a set of preliminary test simulations including different types of calcium currents. Consistent with the results obtained by Zou et al. (2011) the overall cell's excitability did not change significantly, so we decided not to consider *Ca*^++^ channels in our model in this study.

## Results

**Figure 2:**
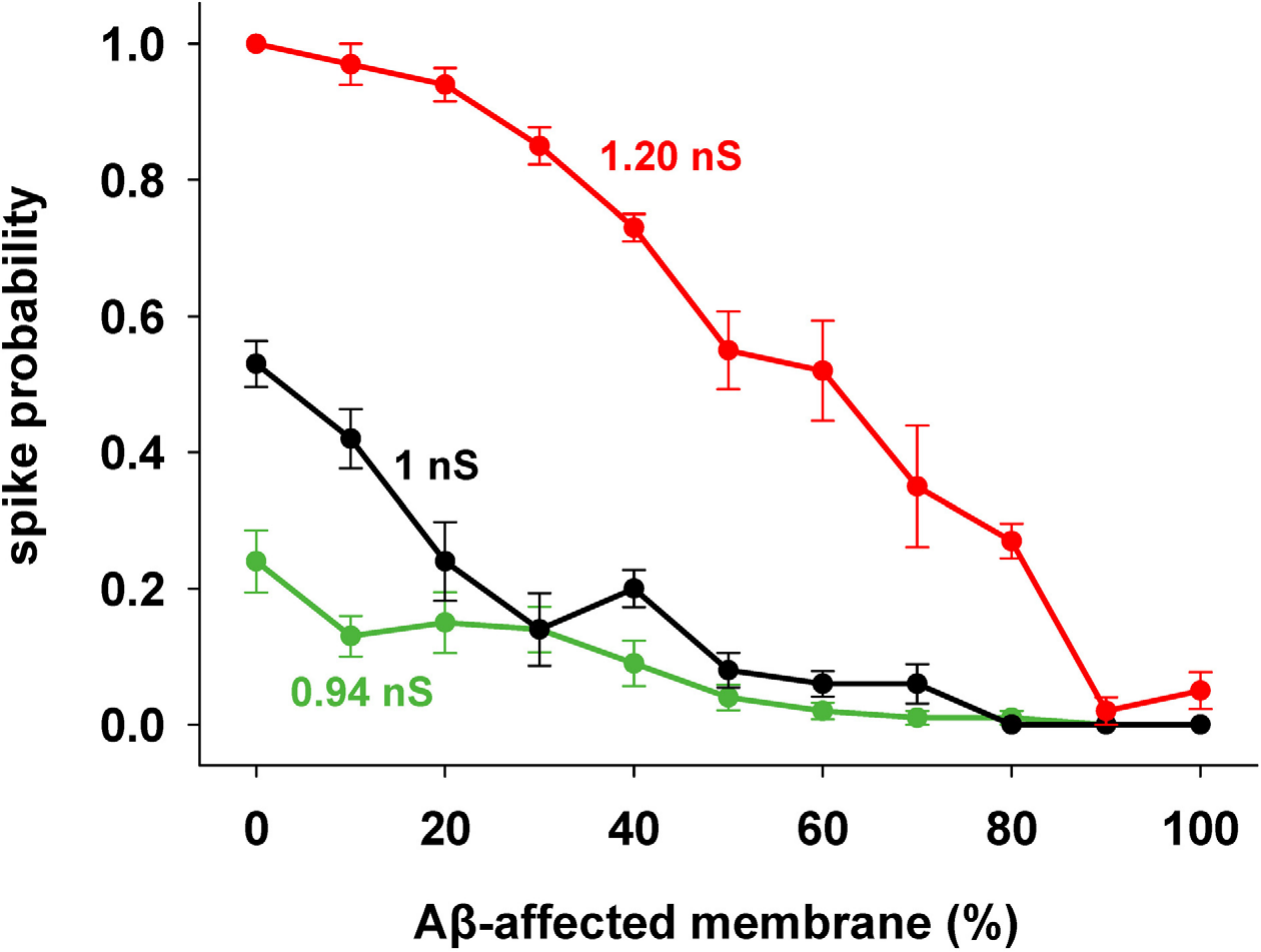
**Progressive effects of Aβ.** Spike probability as a function of the portion of membrane area affected by Aβ, and for three different values of peak synaptic conductance: 0.94 nS (*green*), 1 nS (*black*), and 1.20 nS (*red*). Spike probability (±s.e.m.) was calculated from a set of 100 simulations with random redistribution of synaptic locations, affected dendrites, and synaptic activation times.

We focused our attention on how AD progression can affect the probability of a neuron reaching spike threshold following a synaptic stimulation. Given the low averaging firing rate of these neurons *in vivo* (Csicsvari et al., 1999) individual spikes carry high information content, so this is one of the major factors to test under pathological conditions. Figure 1C shows typical recordings of somatic membrane potential during 10 different simulations, for three different amounts of impaired area (i.e., 30, 60, and 90%) and for 1 nS synapes (0.3 in distal compartments). The neuron's excitability decreases with the amount of membrane affected, when all mechanisms are taken into account. This effect is summarized in Figure 2, where we show the spike probability as a function of the Aβ-affected membrane for three values of synaptic conductance [0.94 nS (*green*), 1.00 nS (*black*), and 1.20 nS (*red*)]. The results show that, independent of synaptic strength, the overall effect of progressive Aβ accumulation on the neuron's membrane is to depress neuronal excitability.

### Contribution of individual currents

**Figure 3:**
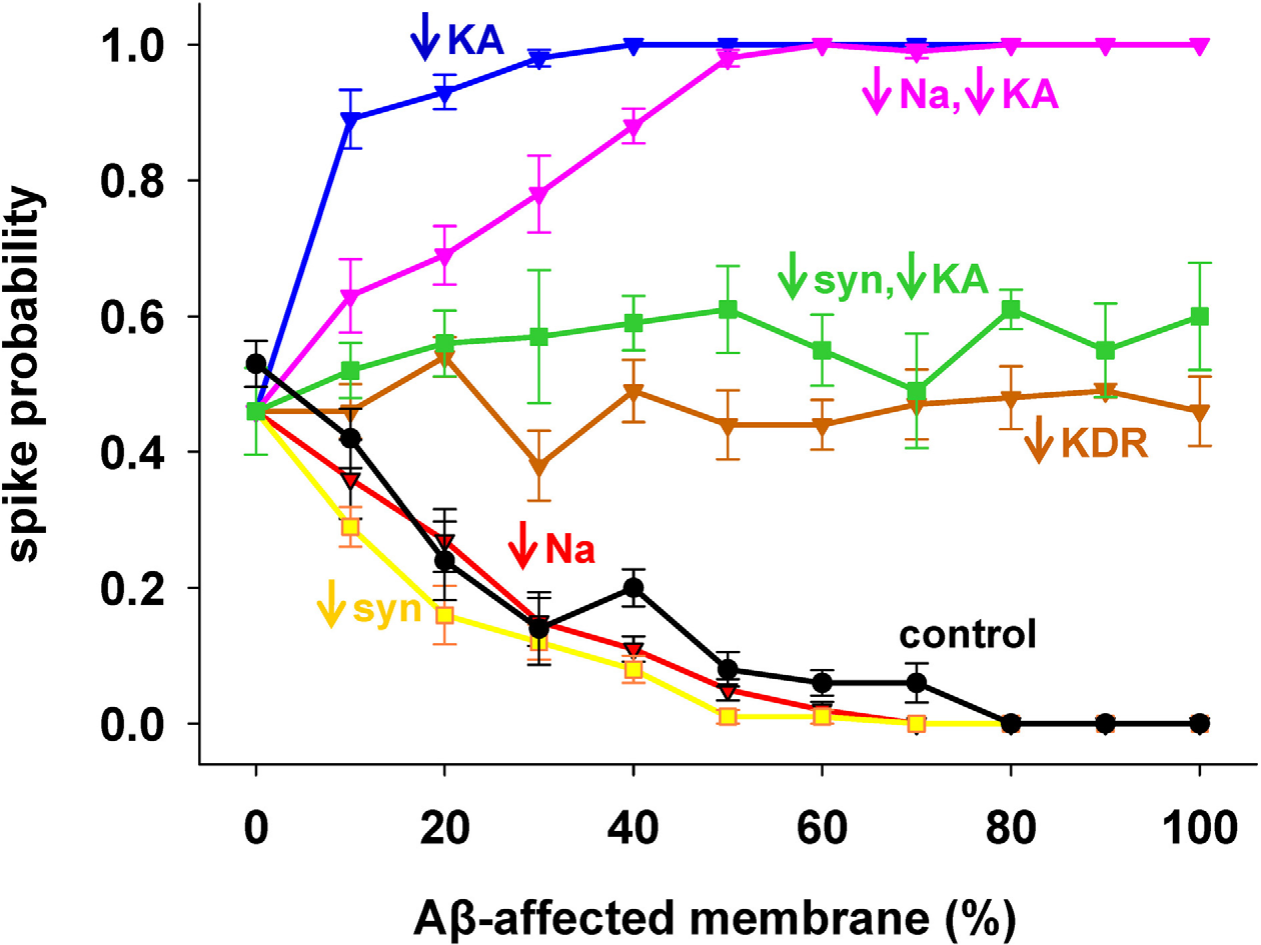
**Individual contribution of membrane mechanisms to the overall effect of Aβ.** Control curve (*black*) is the result obtained, for 1 nS synapses, by the combined effect of Aβ on peak channel conductance [*K*_*A*_ (−60%), *K*_*DR*_ (−40%), *Na* (−50%)], and peak synaptic conductance (−50%); (*blue*) effect of *K*_*A*_ reduction only; (*red*) reduction of *Na* current only; (*pink*) *K*_*A*_ reduction combined with Na reduction; (*yellow*) reduction of synaptic input only; (*brown*) reduction of *K*_*DR*_ only; (*green*) combined reduction of *K*_*A*_ and synaptic conductance.

To evaluate the role of each channel to the overall effect of AD, we started from the results with 1 nS inputs corresponding to a medium stimulation strength that elicited a spike in about 50% of the cases. Results are shown in Figure 3, with pathological conditions (i.e., including all Aβ effects) represented by the *black* curve. The model suggested that the experimentally observed reduction in only the *Na*^+^ current (Figure 3, *red*) or only the synaptic inputs (Figure 3, *yellow*) would account for the whole effect of AD on spike probability (Figure 3, compare *red* and *yellow* with *black*). Instead, a decrease in *K*_*DR*_ current only (Figure 3, *brown*) does not influence the spike probability of a healthy neuron (i.e., it is approximately the same independently of the affected membrane). In striking contrast, and in agreement with a previous model (Morse et al., 2010), by modifying only the *K*_*A*_ current (Figure 3, *blue*) the neuron becomes much more excitable. This excitatory effect persists even when coupled with the *Na*^+^ reduction (Figure 3, *pink*). Interestingly, the simultaneous modification of the synaptic weights and *K*_*A*_ current (Figure 3, *green*) balanced their opposite effects, leading to a spike probability close to the healthy case (i.e., the value at 0% of affected membrane). These results suggest that the pathological modulation of each type of conductance can contribute to the overall effect in different quantitatively and qualitatively ways, and that an appropriate *K*_*A*_ pharmacological manipulation could compensate the negative effects of Aβ on the synaptic inputs.

### *K*_*A*_ pharmacological manipulations as possible treatment of AD effects

**Figure 4:**
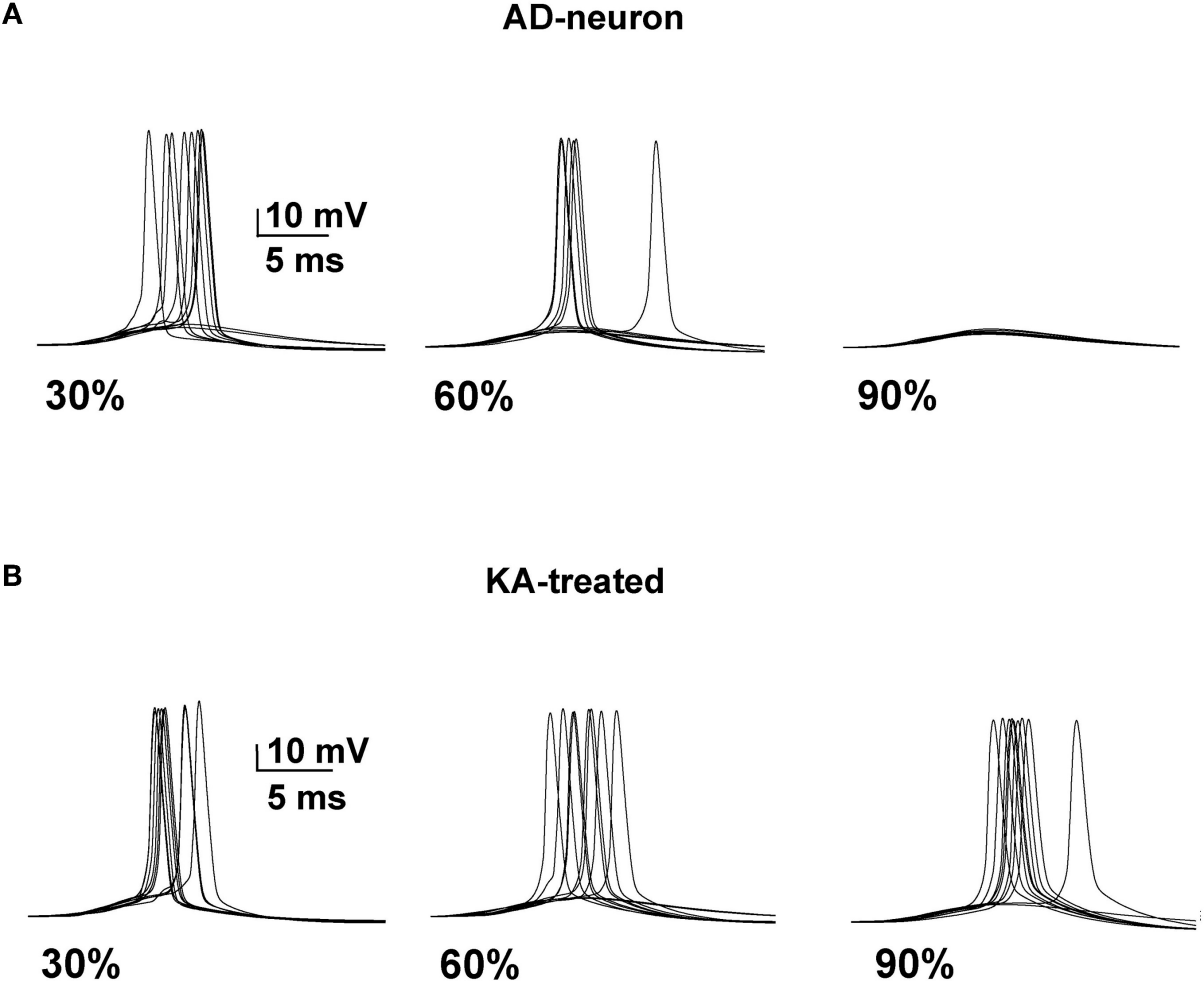
**A decrease of the *K*_*A*_ current counterbalances the effect of AD. (A)** Somatic membrane potential from 10 simulations with different amount of membrane affected by AD. **(B)** Results after a 40% reduction of *K*_*A*_.

We next tested whether it was possible to restore a neuron affected by AD to the spiking probability of a healthy neuron with a drug targeting one or more specific membrane mechanisms. This is possible, in principle, since there are drugs that selectively act on specific channels (discussed in Ferrante et al., 2008). We started from an AD-affected neuron and further reduced the *K*_*A*_ by 40%. In Figure 4 we show typical simulation findings for somatic recordings during 10 simulations using different amounts of affected membrane before and after this “*K*_*A*_ treatment”. As can be seen, in the untreated neuron case (Figure 4, top) the number of spikes decreased with the amount of affected membrane, whereas the same number of APs was obtained in all cases after treatment (Figure 4, bottom). These results demonstrate that it could be possible to manipulate a single type of channel to restore healthy conditions.

**Figure 5:**
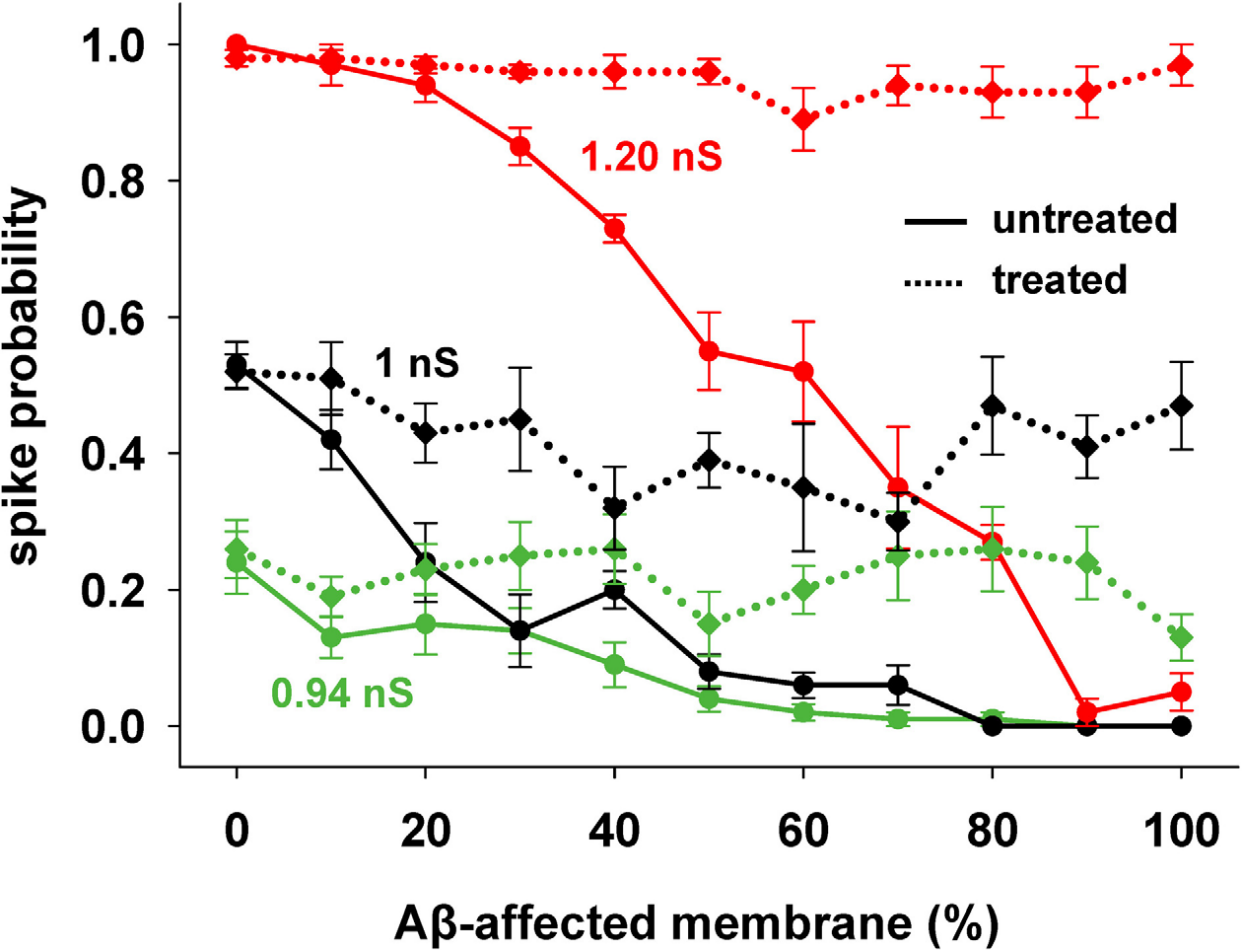
***K*_*A*_ current reduction counterbalances the inhibitory effect of AD in spite of the increasing Aβ accumulation.** Results from AD-neuron (*solid lines*) compared with the results after *K*_*A*_ treatment for three different strengths of synaptic input. Average (±s.e.m.) values calculated from 100 simulations.

This kind of treatment was then tested on the entire range of synaptic input strengths. As shown in Figure 5, a single manipulation of the *K*_*A*_ current (a 40% reduction, on top of the already reduced value caused by AD) was able to obtain a spiking probability that was similar to healthy conditions in all cases (Figure 5, compare solid with dotted lines of the same color).

**Figure 6:**
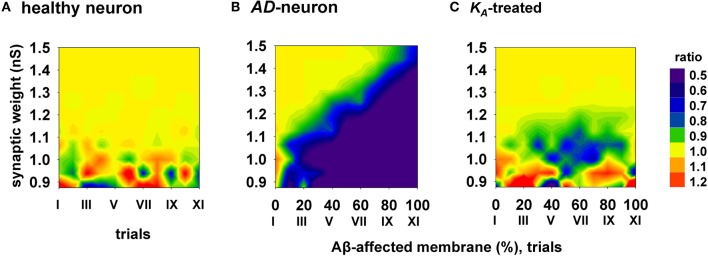
**Overall improvement of a *K*_*A*_-treatment.** All contour plots show, for each condition of synaptic weight and % of impaired surface, the ratio between the average value of spike probability and its value under control conditions (i.e., healthy neuron); healthy condition is represented by a ratio around 1. **(A)** average ratio for 11 trials (differing each other for redistribution of damaged neuronal area, synapses location and activation time) under healthy conditions; **(B)** the ratio is decreased by AD, with a stronger effect for higher portion of damaged membrane; **(C)** after *K*_*A*_-treatment, the ratio is restored to a value around 1 (healthy conditions) for a wide range of parameters.

To evaluate the efficacy and how generalizable this kind of treatment could be, with each combination of synaptic input strength and amount of affected membrane, we calculated the ratio between the spike probabilities obtained under healthy (0% of membrane affected by AD) and damaged membrane conditions (Figure 6). A ratio close to one (Figure 6, yellow areas) is typical of a condition in which the firing properties are not affected by the progression of the disease whereas a low ratio indicates a depressed neuronal activity, typical of AD. The average results for a healthy neuron, grouped in 11 trials (Figure 6, “I” to “XI”, each composed by 100 simulations) are plotted in Figure 6A, and show a ratio of one for most values of synaptic input. The region with scattered colors demarks weaker input stimuli and highlights trial-by-trial fluctuations in the spike probability. For a better comparison of the differences in the firing properties between a healthy and damaged neuron, the results from each of the 11 trials with a healthy neuron were compared with the results obtained with an increasing portion of membrane affected by AD, in 10% increments. As shown in Figure 6B, the ratio between spike probabilities for a neuron affected by AD falls down to zero with the progression of the disease (blue region in Figure 6B). This effect is quite dramatic for weaker synaptic inputs, and less important for stronger inputs. To test whether and to what extent a change in *K*_*A*_ can restore the healthy conditions (i.e., those plotted in Figure 6A), all simulations carried out under AD conditions (Figure 6B) were repeated after a further 40% reduction of *K*_*A*_. The resulting contour plot (Figure 6C) was very similar to healthy conditions. We tested other different manipulations that, in principle, might also give similar results, e.g., increasing/decreasing the time constant of activation/inactivation, but the results were not as profound as the change in the peak conductance (data not shown). These results suggest that a drug targeting the *K*_*A*_ current could be able to balance the depressing effect of AD, in spite of the progression of the disease, maintaining the spike probability close to that obtained in a normal neuron for a wide range of synaptic input strengths.

### Other possible treatments

**Figure 7:**
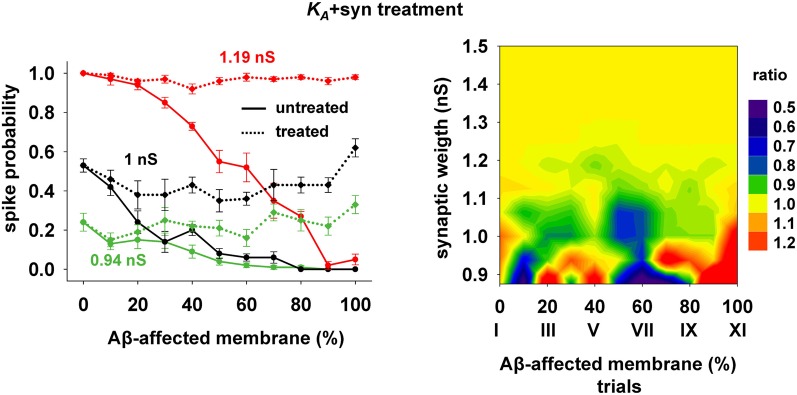
**Overall improvement after increasing the synaptic conductance (+22%) and decreasing the *K*_*A*_ current (−20%).** (*Left*) Spike probability for treated neuron (*dotted lines*) is compared with results from a damaged neuron (*solid lines*), for three different value of synaptic input: 0.94 nS (*green*), 1 nS (*black*), 1.20 nS (*red*). Independently of synaptic strength and increasing impaired surface the original spike probability (control value corresponding to 0% of damaged neuronal area) is restored; *(Right)* Normal conditions (i.e., ratio ≈ 1, yellow area) are obtained for a wide range of synaptic inputs and affected membrane area.

The 40% pharmacological change in *K*_*A*_ discussed in the previous section may seem rather high. We therefore also tested whether the same kind of improvement in firing properties can be obtained with a combination of smaller changes in more than one membrane or synaptic property. We were particularly interested in changes affecting the peak synaptic conductances, because of the plausible link between synaptic plasticity processes at a cellular level and learning and memory at a behavioral level. We found that a simultaneous 20% decrease of *K*_*A*_ and a 22% peak conductance increase of the synapses targeting the membrane compartments affected by AD could give good results. Typical curves for the spike probability and the contour plot for the ratio with healthy conditions are reported in Figure 7. These results show that even a moderate manipulation of *K*_*A*_ could be able, when coupled with a relatively small synaptic potentiation, to restore the firing probability typical of a healthy neuron for a wide range of inputs and the disease's progression.

**Figure 8:**
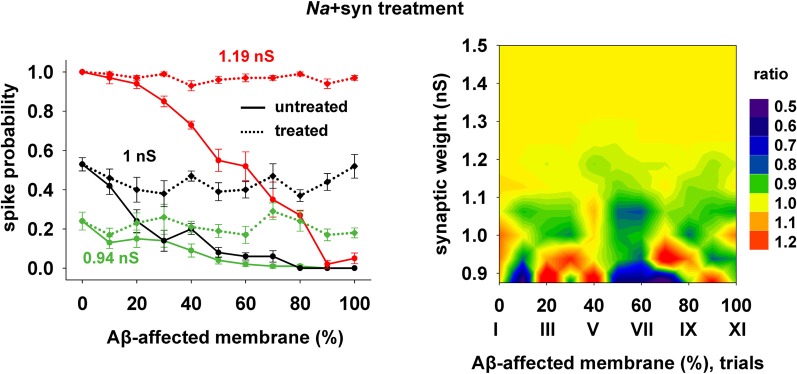
**Overall improvement after increasing the synaptic conductance (+22%) and the *Na* current (+30%).** (*Left*) Spike probability for treated neuron (*dotted lines*) is compared with results from a damaged neuron (*solid lines*), for three different value of synaptic input, as in Figure 7. Independently of synaptic strength and increasing impaired surface, the original spike probability (control value corresponding to 0% of damaged neuronal area) is restored; *(Right)* Normal conditions (i.e., ratio ≈ 1, yellow area) are obtained for a wide range of synaptic inputs and affected membrane area.

Finally, we tested a third treatment using a combination of changes in synaptic strength (+22%) and *Na* conductance (+30%), which could be useful when the disease has affected neurons beyond the CA1 region, and where the *K*_*A*_ may be much lower and its manipulation less effective. The results are plotted in Figure 8, and show that also this kind of change can give good results for a wide range of parameters.

## Discussion

AD is the most prevalent form of dementia, associated with memory deficits and cognitive decline. Several emerging studies (LaFerla et al., 2007) point out that intracellular Aβ-peptides, besides NFT or plaques, play a key role in the pathogenesis of the disease. A number of experimental findings on Aβ effects on ion channels or synaptic properties are available, but each of them is focused on a single mechanism. To the best of our knowledge, there are no experimental or computational studies that simultaneously consider the combined effect of several membrane modifications caused by AD. For example, at the single neuron level, Morse et al. (2010) examined in great detail the effect of *K*_*A*_ reduction (caused by Aβ) on a CA1 pyramidal neuron. The results provided evidence that the thin oblique dendrites in these neurons are the most sensitive to the changes in the backpropagation of an action potential induced by the Aβ-dependent *K*_*A*_ blockade, which may result in an abnormal, and possibly toxic, increase in calcium influx. At the network level, Hasselmo (1997) used a network dynamic model to study the phenomenon of runaway synaptic modification, showing that it is linked to the spreading of neuropathology from the hippocampus into neocortical structures and to memory deficits. Other computational models focused only on synaptic dysfunction (Ruppin and Reggia, 1995; Horn et al., 1996; Rowan, 2012). More related to our work, Zou et al. (2011) investigated the AD-induced theta rhythm abnormalities considering the Aβ effects on four ionic channels (*L*-type *Ca*^2+^, *K*_*A*_, *K*_*DR*_, and *Ca*^2+^-activated *K*^+^ current). However, the simulations carried out included only one channel modification at a time. They showed that only a *K*_*A*_ reduction can induce the typical increase in hippocampal-septal theta band power observed in AD [Ponomareva et al. (2008)], suggesting that a pharmacological action on this current could be a way to reduce the effects of Aβ accumulation.

In our model we considered the combined effect of Aβ on synapses and different ion channels. We were able to show how and which mechanism can be the target of possible drugs for the treatment of AD. The model predicts that a ≈20% increase of *K*_*A*_ or *Na* currents, combined with a similar increase in synaptic conductance, may be sufficient to obtain a significant improvement of the firing probability of a CA1 neuron. The possibility to use *K*_*A*_ channel manipulations in the attempt to ameliorate the effects of AD is particularly intriguing. The peculiar distribution of these channels in CA1 principal neurons (reviewed in Migliore and Shepherd, 2002), expressed at an increasing dendritic density with distance from the soma, suggests that a drug acting on *K*_*A*_ would be most effective only in these neurons, thus with limited collateral effects in other brain regions. On the contrary, a drug acting on *Na* channels, which have a much wider distribution (Migliore and Shepherd, 2002), could be more effective when the disease has also spread in other brain regions. The effect of increasing the synaptic weight should also be stressed. It corresponds to the physiological Long-Term Potentiation mechanism, and it can be obtained by just “using” the synapses by refreshing old memories to delay the effects of the degenerative disease. This is consistent with experimental findings (Billings et al., 2007) suggesting that learning slows the development of two brain lesions related to AD. A conceptually similar result was also obtained by Bentwich et al. (2011) with transcranial magnetic stimulation combined with cognitive training.

A possible limitation of our approach is that Aβ-dependent changes may initiate compensatory mechanisms involving ionic and synaptic currents through homeostatic mechanisms that could, in part, be responsible for the observed neuron properties (e.g., Horn et al., 1996; MacLean et al., 2005). Additional processes not considered in this work, such as the effects of Aβ on the interneurons responsible for feedforward inhibition, may contribute to the overall effect on the spike threshold. However, there is not enough information on the nature and extent of these mechanisms in the current experimental models of AD to implement a realistic model for them. A more detailed analysis of their actual role was thus outside the scope of this paper.

In conclusion, our model allowed us to take into account different experimental findings on the effects of Aβ accumulation, providing new insight on how the combined interaction of different mechanisms can lead to overall effects that may be closer to what happens *in vivo*. The model predictions on the possible pharmacological treatments can be readily tested experimentally. As new and more detailed experimental data will be available, new, and more specific actions can be modeled and tested to facilitate the long and expensive experimental investigations that are currently and strongly limiting the development of new drugs in this field.

### Conflict of interest statement

The authors declare that the research was conducted in the absence of any commercial or financial relationships that could be construed as a potential conflict of interest.
